# Presence of an Artificial Intelligence–powered Predictive Biomarker Is Associated with a Poor Response to Intravesical Bacillus Calmette-Guerin but Not to Intravesical Sequential Gemcitabine/Docetaxel in Patients with High-grade Non–muscle-invasive Bladder Cancer

**DOI:** 10.1016/j.euo.2025.04.006

**Published:** 2025-04-25

**Authors:** Vignesh T. Packiam, Ian M. McElree, Saum Ghodoussipour, Vivek Nimgaonkar, Viswesh Krishna, Joon Kyung Kim, Derek B. Allison, Jordan R. Richards, K.D. Anand Rajan, Stephanie J. Chen, Yair Lotan, Stephen B. Williams, Haochen Zhang, Drew Watson, Damir Vrabac, Waleed M. Abuzeid, Anirudh Joshi, Ashish M. Kamat, Michael A. O’Donnell, Patrick J. Hensley

**Affiliations:** a Department of Urology, Rutgers Cancer Institute, New Brunswick, NJ, USA; b Department of Urology, University of Iowa, Iowa City, IA, USA; c Valar Labs, Palo Alto, CA, USA; d Department of Urology, University of Kentucky College of Medicine, Lexington, KY, USA; e Department of Pathology, University of Kentucky College of Medicine, Lexington, KY, USA; f Department of Pathology, University of Iowa, Iowa City, IA, USA; g Department of Urology, University of Texas Southwestern Medical Center, Dallas, TX, USA; h Department of Urology, University of Texas Medical Branch, Galveston, TX, USA; i Department of Urology, MD Anderson Cancer Center, Houston, TX, USA

**Keywords:** Artificial intelligence, Bacillus Calmette-Guerin, Biomarker, Docetaxel, Gemcitabine, Machine learning, Non–muscle-invasive bladder cancer

## Abstract

Intravesical bacillus Calmette-Guerin (BCG) is considered first-line adjuvant therapy for high-risk or high-grade non–muscle-invasive bladder cancer (NMIBC). Recently, sequential intravesical gemcitabine and docetaxel (Gem/Doce) has emerged as a promising alternative to intravesical BCG. Biomarkers to select the optimal treatment regimen could facilitate clinical decision-making. The Computational Histologic Artificial Intelligence (CHAI) platform was previously used to develop an artificial intelligence–augmented histologic assay (CHAI biomarker) that identified patients with NMIBC at an increased risk of recurrence and progression events following BCG treatment. In this study, we assessed use of the CHAI biomarker among patients with treatment-naive high-grade NMIBC who received intravesical BCG or Gem/Doce. Among patients with the presence of the CHAI biomarker, those treated with BCG had a 24-mo high-grade recurrence-free survival (HG-RFS) rate of 56% (95% confidence interval [CI] 43–73%) and those treated with Gem/Doce had an HG-RFS rate of 90% (95% CI 79–100%; hazard ratio [HR] 5.4, 95% CI 1.6–18.3, *p* = 0.007). Among patients with an absence of the CHAI biomarker, those treated with BCG or Gem/Doce had no significant difference in HG-RFS (HR 1.3, 95% CI 0.6–2.6, *p* = 0.5). The interaction term between the CHAI biomarker and the treatment type was significant (*p* = 0.029), indicating an association between the biomarker and the clinical outcome that is dependent on the treatment received. This study suggests that the CHAI biomarker predicts which specific high-grade NMIBC patients are less likely to benefit from BCG and may benefit from alternative treatments including, potentially, Gem/Doce.

Intravesical bacillus Calmette-Guerin (BCG) is the preferred adjuvant therapy to prevent recurrence and progression of high-grade non–muscle invasive bladder cancer (NMIBC) [1]. While level 1 data support BCG over single-agent chemotherapy, the global BCG shortage has spurred investigation of alternative regimens. Sequential intravesical gemcitabine and docetaxel (Gem/Doce) has favorable safety and efficacy in NMIBC, and appears similar to BCG for treatment-naive disease in retrospective and prospective analyses [2–5]. The regimen is increasingly used for NMIBC, and the ongoing ECOG-ACRIN EA8212 (BRIDGE) trial is evaluating randomized BCG versus Gem/Doce for NMIBC [6]. Thus, biomarkers to predict a response to BCG versus Gem/Doce can have an immediate impact.

A recent multi-institutional analysis of 944 patients with high-grade NMIBC treated with BCG demonstrated that a signature of histologic features analyzed with the Computational Histologic Artificial Intelligence (CHAI) platform in conjunction with clinical factors identified patients at high risk of recurrence, progression, and development of BCG-unresponsive disease [7]. However, it is unclear whether the CHAI biomarker is prognostic for adverse oncologic outcomes to any treatment or whether it can predict a response specifically to BCG versus alternative therapies. In this study, we assessed whether the CHAI biomarker was associated with a response to treatment with BCG and, similarly, whether CHAI biomarker status was associated with a response to treatment with Gem/Doce in patients with high-grade NMIBC. Causality was not assessed in this study.

This study was approved by the University of Iowa and University of Kentucky institutional review boards. Consecutive patients with treatment-naive high-grade NMIBC and available pretreatment transurethral resection of bladder tumor–derived whole slide images (WSIs), who received adjuvant intravesical BCG or Gem/Doce between 2011 and 2021 (Iowa) or 2008 and 2023 (Kentucky), were included. Patients received both induction and maintenance treatments as described previously [2]. All BCG-treated patients received at least 1 yr of maintenance therapy unless interrupted by BCG shortage. Patient characteristics were not considered in treatment allocation, which was primarily based on the availability of BCG at the time of treatment commencement. None of the included patients were used in the development of the CHAI biomarker, and the entire cohort was used as an external validation set. WSIs of hematoxylin and eosin–stained slides were analyzed using the CHAI platform for the risk of high-grade recurrence and labeled as the CHAI biomarker present or absent using the validated locked histologic feature model described previously [7]. Multifocality was removed from the model given its prognostic effect [8].

Within each treatment group, Fisher’s exact test (for categorical variables) and Wilcoxon rank sum test (for continuous variables) were used to test whether baseline clinicopathologic characteristics had different distributions between the biomarker and treatment groups. Multivariable Cox proportional hazard (CPH) models for high-grade recurrence-free survival (HG-RFS) and likelihood ratio tests were used to test the biomarker-treatment interaction, including American Urological Association (AUA) and European Association of Urology (EAU) risk groups as independent variables. Multivariable CPH regression, including AUA risk stratification (intermediate/high) as an independent variable, was performed to compare HG-RFS between and within the biomarker present and absent groups. The Kaplan-Meier method was used to estimate HG-RFS in BCG- and Gem/Doce-treated cases stratified further by biomarker status. A p value of <0.05 was considered significant.

The study included 253 patients, of whom 159 (63%) received BCG and 94 (37%) received Gem/Doce (Table 1). There were no differences in patient or tumor characteristics between the Gem/Doce- and BCG-treated cohorts with the exception of follow-up duration (Supplementary Table 1). The median follow-up was 37 mo (interquartile range [IQR] 18–58) for all patients and 36 mo (IQR 18, 59) for patients without any high-grade recurrence. In 77 (30%) patients, the CHAI biomarker was present, while in 176 (70%) the biomarker was absent. Among patients with the biomarker present, 44 received BCG (57%) and 33 received Gem/Doce (43%). Demographic and clinical tumor characteristics did not vary by the presence of the CHAI biomarker, with the exception of an association between the biomarker presence/absence and EAU risk groups in BCG-treated patients (*p* = 0.021; Table 1). Among BCG-treated patients, those with the biomarker had inferior HG-RFS to those without the biomarker (hazard ratio [HR] 2.0, 95% confidence interval [CI] 1.1–3.6, *p* = 0.023), and in Gem/Doce-treated patients, there was no significant difference in HG-RFS between those with and without the biomarker (HR 0.47, 95% CI 0.13–1.7, *p* = 0.2; Fig. 1 and Supplementary Fig. 1). Among the patients with the CHAI biomarker present, the 24-mo HG-RFS rates were 56% (95% CI 43–73%) in BCG-treated cases and 90% (95% CI 79–100%) in Gem/Doce-treated patients (HR 5.4, 95% CI 1.6–18.3, *p* = 0.007; Fig. 1). Among the patients in whom the biomarker was absent, BCG- and Gem/Doce-treated HG-RFS outcomes were not significantly different (HR 1.3, 95% CI 0.6–2.6, *p* = 0.5; Fig. 1). Using CPH regression models, the likelihood ratio test for the biomarker-treatment interaction term was significant (*p* = 0.029). The HG-RFS results for the biomarker-treatment interaction were maintained after adjusting for the EAU and AUA risk groups (*p* = 0.029 for both).

To our knowledge, this study is the first to evaluate an artificial intelligence (AI)-based histologic biomarker to predict differences in response to different intravesical therapies among patients with treatment-naive high-grade NMIBC. Consistent with the prior report, the previously validated CHAI biomarker identifies NMIBC patients with an inferior response to BCG [7]. The presence of the CHAI biomarker was associated with poorer HG-RFS in patients treated with BCG but was not associated with poorer HG-RFS in Gem/Doce-treated patients. The biomarker-treatment interaction term was significant, indicating that the biomarker predicts treatment outcome and is dependent on the treatment received.

The recent development of promising therapies for NMIBC has been a boon for patients and physicians [9]. There is an unmet need for the prediction of a response to therapies. In the treatment-naive setting for high-grade NMIBC, BCG is the ‘‘gold standard’’ as per the treatment guidelines published by the AUA and the EAU [1,10]. However, the ongoing international shortage has limited patient access to BCG. While there are reports of prognostic biomarkers for BCG, no predictive risk factors have been described that facilitate selection of first-line treatment for NMIBC. There is increasing evidence supporting the viability of intravesical Gem/Doce as an alternative to BCG, and there is biologic plausibility to identifying histologic characteristics to predict a response. Notably, there is a logarithmic attrition of a response to immunotherapies such as BCG due to the inability to mount an effective immune response early after treatment, in contrast to the linear recurrence that generally occurs after chemotherapy [2]. The CHAI biomarker represents an advance toward personalized medicine and can identify poor responders to BCG, sparing ineffective treatment in patients unlikely to benefit and prioritizing limited BCG supplies for patients most likely to benefit.

There are limitations to this study. It is retrospective, and the cohort was obtained from two sites. The nonrandomized design lends itself to unknown confounders potentially influencing treatment allocation. The wide CIs are secondary to the smaller proportion of patients in the category with the biomarker present. Within the study cohort, patients treated with Gem/Doce had favorable HG-RFS compared with those treated with BCG, and a study of this finding is ongoing in a randomized controlled trial of Gem/Doce versus BCG [6]. Further clinical validation of this biomarker will ideally involve an analysis of a prospectively accrued cohort.

In conclusion, our study reports the first AI-powered histologic biomarker for patients with untreated high-grade NMIBC that allows for effective prediction of poor responders to BCG but does not stratify patient response in those treated with Gem/Doce. This suggests that in patients who are predicted as poor responders to BCG, alternative treatments—potentially including Gem/Doce—could be considered. These findings begin to offer an avenue for precision medicine in the treatment of this disease.

## Supplementary Material

Supplemental Materials

S1 MMC3

S1 MMC1

S1 MMC2

Appendix A. Supplementary material

Supplementary data to this article can be found online at https://doi.org/10.1016/j.euo.2025.04.006.

## Figures and Tables

**Fig. 1 –:**
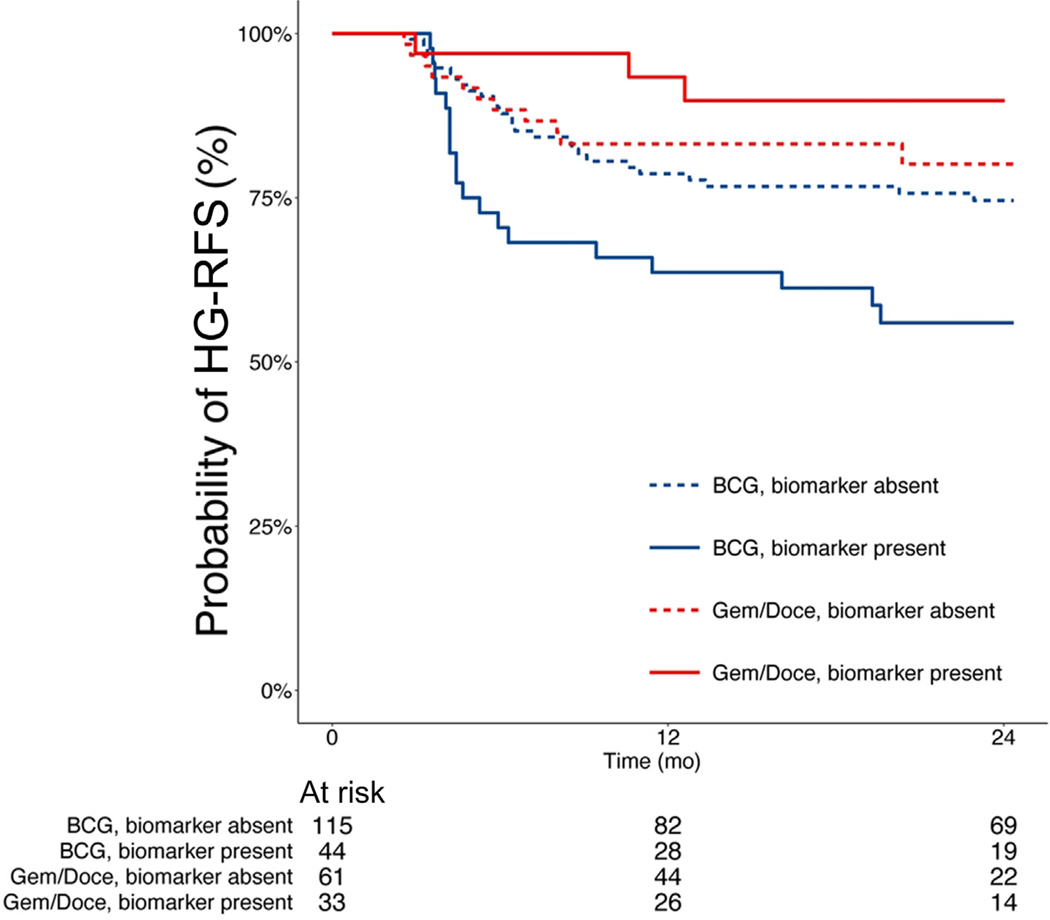
High-grade recurrence free survival stratified by treatment and CHAI biomarker status. Kaplan-Meier estimates for HG-RFS are reported for patients stratified by treatment and biomarker status. Among BCG-treated patients, patients with the CHAI biomarker present performed worse than those with the CHAI biomarker absent (HR 2.0, 95% CI 1.1–3.6, *p* = 0.023). Among Gem/Doce-treated patients, there was no significant difference in HG-RFS based on biomarker status (HR 0.47, 95% CI 0.13–1.7, *p* = 0.2). Presence of the CHAI biomarker was associated with worse HG-RFS in BCG-treated patients but not in patients treated with Gem/Doce (HR 5.4, 95% CI 1.6–18.3, *p* = 0.007). Significant differences in HG-RFS were not seen in patients with the CHAI biomarker absent who were treated with either BCG or Gem/Doce (HR 1.3, 95% CI 0.6–2.6, *p* = 0.5). BCG = bacillus Calmette-Guerin; CHAI = Computational Histologic Artificial Intelligence; CI = confidence interval; Gem/Doce = gemcitabine and docetaxel; HG-RFS = high-grade recurrence-free survival; HR = hazard ratio.

**Table 1 –:** Patient characteristics by treatment received and CHAI biomarker status

Characteristic	Gem/Doce			BCG		
		
	Biomarker absent (*N* = 61)	Biomarker present (*N* = 33)	*p* value	Biomarker absent (*N* = 115)	Biomarker present (*N* = 44)	*p* value

Median age (Q1, Q3)	73 (66, 81)	73 (66, 79)	0.6 ^a^	73 (64, 80)	76.0 (70, 83)	0.11 ^a^
Sex, *n* (%)			0.4^2^			0.7^2^
Female	13 (21)	4 (12)		24 (21)	7 (16)	
Race, *n* (%)			>0.9 ^b^			0.3 ^b^
Non-White	3 (4.9)	1 (3.0)		10 (8.7)	1 (2.3)	
Site, *n* (%)			>0.9 ^b^			0.4 ^b^
Iowa	49 (80)	27 (82)		78 (68)	33 (75)	
Kentucky	12 (20)	6 (18)		37 (32)	11 (25)	
Smoking status, *n* (%)			0.4 ^b^			0.3 ^b^
Current	29 (48)	21 (64)		68 (59)	20 (45)	
Former	13 (21)	5 (15)		19 (17)	9 (20)	
Never	19 (31)	7 (21)		28 (24)	15 (34)	
Pretreatment tumor pathology, *n* (%)			0.12 ^b^			0.5 ^b^
CIS alone	7 (11)	0		10 (8.7)	2 (4.5)	
T1	17 (28)	16 (48)		46 (40)	24 (55)	
T1 + CIS	8 (13)	5 (15)		11 (9.6)	5 (11)	
Ta	19 (31)	7 (21)		36 (31)	11 (25)	
Ta + CIS	10 (16)	5 (15)		12 (10)	2 (4.5)	
Pretreatment tumor size (cm), *n* (%)			0.2 ^b^			0.8 ^b^
≤3	22 (36)	14 (42)		37 (32)	14 (32)	
>3	22 (36)	15 (45)		60 (52)	25 (57)	
Unknown	17 (28)	4 (12)		18 (16)	5 (11)	
Pretreatment CIS-containing tumor, *n* (%)	25 (41)	10 (30)	0.4 ^b^	33 (29)	9 (20)	0.3 ^b^
Pretreatment multifocal, *n* (%)	17 (28)	12 (36)	0.5 ^b^	30 (26)	15 (34)	0.3 ^b^
Pretreatment variant histology, *n* (%)	7 (11)	3 (9.1)	>0.9 ^b^	8 (7.0)	4 (9.1)	0.7 ^b^
AUA risk group, *n* (%)			0.4 ^b^			0.3 ^b^
High	56 (92)	32 (97)		105 (91)	43 (98)	
Intermediate	5 (8.2)	1 (3.0)		10 (8.7)	1 (2.3)	
EAU risk group, *n* (%)			0.5 ^b^			0.021 ^b^
Intermediate	9 (15)	2 (6.1)		25 (22)	2 (4.5)	
High	43 (70)	26 (79)		77 (67)	35 (80)	
Very high	9 (15)	5 (15)		13 (11)	7 (16)	
Median time to last follow-up or death (mo; Q1, Q3)	23 (14, 39)	22 (16, 35)	0.9 ^a^	50 (27, 78)	48 (24, 78)	0.9 ^a^

AUA = American Urological Association; BCG = bacillus Calmette-Guerin; CHAI = Computational Histologic Artificial Intelligence; CIS = carcinoma in situ; EAU = European Association of Urology; Gem/Doce = gemcitabine and docetaxel.

Tabulated patient characteristics are reported among patients treated with Gem/Doce and BCG and, within each of these groups, between patients with the CHAI biomarker present and absent. Comparisons were made using the Fisher’s exact test for categorical variables and Wilcoxon rank sum test for continuous variables.

a Wilcoxon rank sum test.

b Fisher’s exact test.

## ADVANCING PRACTICE

### What does this study add?

Intravesical bacillus Calmette-Guerin (BCG) and, more recently, sequential intravesical gemcitabine and docetaxel (Gem/Doce) are used as adjuvant therapy for high-grade non–muscle-invasive bladder cancer (NMIBC), but there is no reliable tool for selecting the optimal treatment plan. In this study, we validated an artificial intelligence–augmented histologic assay—termed the Computational Histologic Artificial Intelligence (CHAI) biomarker—in treatment-naïve patients with high-grade NMIBC who received adjuvant intravesical BCG or Gem/Doce. Among patients with the presence of the CHAI biomarker, those treated with BCG had a 24-mo high-grade recurrence-free survival (HG-RFS) rate of 56% and those treated with Gem/Doce had an HG-RFS rate of 90% (hazard ratio [HR] 5.4, *p* = 0.007). Among patients with an absence of the CHAI biomarker, those treated with BCG or Gem/Doce had no significant difference in HG-RFS (HR 1.3, *p* = 0.5). Critically, the test of interaction between the CHAI biomarker and the treatment type was statistically significant, indicating that this artificial intelligence–powered biomarker is the first to effectively predict which patients with high-grade NMIBC are less likely to benefit from BCG and may benefit from alternative treatments including Gem/Doce.

### Clinical Relevance

Non-muscle-invasive bladder cancer is a heterogeneous disease and there has been a long search for reliable biomarkers that help guide treatment selection. In this study, the authors evaluated the Computational Histology Artificial Intelligence (CHAI) biomarker status and determined whether there was any association with treatment response towards intravesical BCG and sequential intravesical gemcitabine and docetaxel (Gem/Doce). Among the 253 patients being included, 63% received BCG and 37% received Gem/Doce. The authors found that CHAI biomarker positive status was associated with worse treatment response towards BCG, but not towards Gem/Doce. While such findings still require stringent prospective validation, this could represent an important step incorporating artificial intelligence and biomarkers into our treatment algorithm. Associate Editor: Jeremy Y.C. Teoh, M.D.

### Patient Summary

Treating non–muscle-invasive bladder cancer, after surgical removal of the tumor, with bacillus Calmette-Guerin (BCG) in the bladder reduces the chance of the cancer returning. More recently, the use of two chemotherapies, gemcitabine and docetaxel (Gem/Doce), has also been shown to be effective. We found that an artificial intelligence–powered biomarker identifies patients who have poorer outcomes when treated with BCG, but it is not associated with a poor response among patients treated with Gem/Doce, suggesting that this tool could be used to identify patients in whom alternative treatments can be considered.
