# Supplementary material for: Presence of an Artificial Intelligence–powered Predictive Biomarker Is Associated with a Poor Response to Intravesical Bacillus Calmette-Guerin but Not to Intravesical Sequential Gemcitabine/Docetaxel in Patients with High-grade Non–muscle-invasive Bladder Cancer
Source: Eur Urol Oncol. Author manuscript; Available in PMC 2026 Feb 16. (PMC12907750; doi:10.1016/j.euo.2025.04.006)
Supplement: Supplemental Materials [file NIHMS2110211-supplement-Supplemental_Materials.zip › 1-s2.0-S2588931125001002-mmc1.docx]

**Supplementary material**

**Supplementary methods**

***Development of artificial intelligence–based histologic biomarkers using the Correlative Histology Artificial Intelligence platform***

The development of the artificial intelligence (AI)-based histologic biomarker using the Correlative Histology Artificial Intelligence (CHAI) platform followed the methodology described previously in a prior publication [1]. In brief, scanned whole-slide images were analyzed through an imaging pipeline consisting of tissue segmentation and nuclei segmentation deep learning AI models. To create an annotated dataset for the AI models, 512 × 512 μm patches were annotated by board-certified pathologists from a selected cohort of representative slides from each of the centers in the development cohort. Patches were annotated for either tissue segmentation or nuclei segmentation. Tissue regions were segmented into bladder papillary tumor, carcinoma in situ, invasive tumor, variant histology, stroma, detrusor muscle, slide background, blood, necrosis, and artifact (consisting of tissue folds, blurring, and other preparation artifacts) categories. Cells were segmented and classified into neoplastic, mitosis, apoptosis, stromal, endothelial, muscle, lymphocyte, eosinophil, neutrophil, macrophage, and plasma categories. All cells that could not be annotated were classified into the “unlabeled” category and were not used for analysis. Patches were split 80:20 for development and internal validation, with the internal validation set being exclusively selected from centers not included in the external validation (EV) cohort used to test generalization of the AI models. The tissue and nuclei segmentation models were initialized with an Imagenet-pretrained ResNet-50 backbone and trained with cross-entropy loss with balanced-batch sampling for 30 epochs with a learning rate of 1e-5 and the AdamW optimizer [2,3].

The EV cohort was composed of centers not included during development to increase the generalizability and clinical interpretability of results. Features were defined based on previous studies with the CHAI platform and a broad literature review, and were grouped into categories representing the hallmarks of cancer, including tumor aggressiveness, tumor proliferation, angiogenesis, invasive features, immune features, and stromal features [4,5]. These feature groups were tested against endpoints using chunk tests to select associated groups, and univariate tests were run for all features in selected groups against endpoints to select the features most associated with the endpoints from each selected group. The final set of screened features were combined with selected clinical variables associated with the clinical endpoints—multifocality and T1 stage for high-grade (HG) recurrence-free survival (RFS) and progression-free survival (PFS), respectively—and were used to construct the linear AI models. Both clinical variables—multifocality for HG-RFS and T1 stage for PFS—added predictive values in addition to the AI features using likelihood ratio tests (multifocality *p* = 0.01, T1 stage *p* = 0.035). Bootstrapping was used to estimate model optimism and measure the bias.

***Statistical analysis***

For model development purposes, risk-based models with continuous linear predictors of clinical risk were derived from multivariable Cox proportional hazard regression models involving AI-based histologic features strongly associated with HG-RFS, PFS, and time to BCG unresponsiveness. Separate clinical thresholds for each linear predictor were used to create corresponding low- and high-risk groups for each clinical endpoint. Owing to the presence of missing multifocality data (6.3% missing at random), to avoid a potential bias and to improve the efficiency of Cox regression estimates, multiple imputation methods based on additive regression, bootstrapping, and predictive mean matching were used. The aregImpute function from the RMS package was used with age, sex, smoking status, T1 stage, T stage, multifocality, variant histology, prior history of recurrence, size of tumor, number of tumors, BCG-unresponsive disease, and high-grade recurrence at 2 yr for 40 imputations. In order to fit the multivariable Cox proportional hazard model, the fit.mult.impute function in the RMS package was used with the predefined imputation variable list. Multivariable Cox proportional hazard regression analyses and likelihood ratio tests were performed to test the hypothesis that the AI-based model consisting of AI features combined with clinical variables (multifocality for HG-RFS and T1 stage for PFS) provided predictive values above and beyond the AI-based models consisting of AI features alone. A prespecified *p* value of <0.05 for the corresponding likelihood ratio tests was considered significant. For the sample size determination for the development and EV cohorts, the sample size procedures for the Cox proportional hazard regression model provided by Hsieh and Lavori [6] was used.

The resulting predefined AI-based models were prospectively validated in the independent EV cohort of 641 patients. For clinical validation purposes, analyses were performed to identify clinical differences between the development and the independent EV study cohorts. For this purpose, Wilcoxon rank-sum tests for continuous variables and chi-square test or Fisher’s exact test in the presence of low cell counts for categorical variables were performed. The performance of each predefined AI-based model was assessed using multivariable Cox proportional hazard regression models and the corresponding likelihood ratio χ² statistics to test the null hypothesis that the hazard ratio for each (binary) AI model was not equal to 1. A prespecified *p* value of <0.05 for the corresponding likelihood ratio tests was considered significant. Additionally, multivariable Cox proportional hazard regression analyses and likelihood ratio tests were performed to test the hypothesis that the AI-based models provided predictive values above and beyond the standard clinical covariates and the European Organization for Research and Treatment of Cancer (EORTC) 2016 and European Association of Urology (EAU) 2021 risk models. A multivariable Cox proportional hazard regression analysis incorporating six clinical variables (age, sex, smoking, T stage, presence of carcinoma in situ, and focality) was used to evaluate the association of these variables with the HG-RFS and PFS endpoints. Again, a prespecified *p* value of <0.05 for the corresponding likelihood ratio tests was considered significant. Forest plots of the estimated hazard ratios and corresponding 95% confidence intervals for each covariate are provided. For completeness, plots of the nonparametric maximum likelihood Kaplan-Meier estimates of survival stratified by the predefined low- and high-risk groups and associated log-rank tests for each clinical endpoint are provided. RFS and PFS endpoints were censored at 2 and 5 yr, respectively.

The AI-based model for PFS was also utilized for the prediction of time to cystectomy. No adjustment for multiplicity of testing the HG-RFS, PFS, time to cystectomy, and time to BCG unresponsiveness clinical endpoints was made. In the EV cohort, missing data were imputed only to allow for the application of the EORTC 2016 risk model (number of tumors and history of recurrence) and EAU 2021 risk model (history of recurrence, size of tumor, and focality). Frank Harrell’s RMS package for R was used for imputation. To avoid a potential bias and improve the efficiency of Cox regression estimates, multiple imputation methods based on additive regression, bootstrapping, and predictive mean matching were used to impute values for missing data. The imputation variables listed above were used to impute the data, and EORTC and EAU scores were calculated across each of the imputed datasets and averaged to produce the final score for an analysis.

Model calibration was assessed using calibration intercept and calibration slope, and calibration of AI-based models was compared with that of the EORTC and EAU risk calculators for descriptive purposes. A complete case analysis using case-wise deletion was also performed for comparative purposes.

In the current study, we tested the hypothesis that the CHAI biomarker is associated with treatment benefit, which requires a comparison of treatment outcomes related to treatment with BCG and gemcitabine/docetaxel. In statistical terms, we must demonstrate that there is a significant interaction between the CHAI biomarker and treatment. For this purpose, we compared the (full) Cox proportional hazard model of HG-RFS as a function of the (continuous) CHAI output, treatment (gemcitabine/docetaxel vs BCG), and the interaction of the CHAI output and treatment versus the (reduced) Cox proportional hazard model, excluding the interaction of the CHAI output and treatment.

A statistical analysis was performed with R v4.2.0 (R Foundation, Vienna, Austria).

**Supplementary results**

***Sample size calculation***

For the development cohort, based on the formula for sample size determination for the Cox proportional hazard model provided by Hsieh and Lavori [6], at a 5% significance level and an event rate of 25%, a Cox regression of the log hazard ratio for the AI predictor with a standard deviation of 0.5 based on a sample of 299 patients achieves 85% power. For the EV cohort, based on the formula for sample size determination for the Cox proportional hazard model provided by Hsieh and Lavori [6], at a 5% significance level and an event rate of 20%, a Cox regression of the log hazard ratio for the AI predictor with a standard deviation of 0.5 based on a sample of 438 patients achieves 90% power. Sample size determinations were based on an assumption that the estimated hazard ratio for the AI predictor exceeds 2, which has been observed in prior studies.

***Calibration results***

The calibration of the AI models was compared with the EORTC and EAU risk calculators using the calibration intercept and slope. The EORTC model for the RFS endpoint evaluated at 12 mo had a calibration intercept of –0.36 and calibration slope of 0.38, compared with the AI model that had a calibration intercept of 0.25 and calibration slope of 0.65. The EAU model for the PFS endpoint evaluated at 24 mo had a calibration intercept of 1.48 and calibration slope of 1.71, compared with the AI model that had a calibration intercept of –0.69 and calibration slope of 0.56.

***Complete case analysis***

For comparative purposes, a complete case analysis was additionally performed to evaluate the additive predictive value of the AI models above the EORTC and EAU risk calculators based on multivariable Cox proportional hazard regression and likelihood ratio tests. For the RFS endpoint subset to complete cases, the AI model provided an added predictive value beyond that of the EORTC model (*p* < 0.001). For the PFS endpoint subset to complete cases, the AI model provided an added predictive value beyond that of the EAU model (*p* = 0.01).

***Demonstration of the prediction of treatment outcome using the CHAI biomarker***

The likelihood ratio statistic resulting from the test of interaction between the CHAI biomarker and treatment was significant (*p* = 0.006), indicating that the CHAI biomarker is also predictive of a treatment benefit from BCG and gemcitabine/docetaxel. The observation that the CHAI biomarker is predictive of a treatment benefit is illustrated through a comparison of the resulting survival distributions stratified by treatment obtained through Kaplan-Meier estimation (Fig. 1).

**References**

[1] Lotan Y, Krishna V, Abuzeid WM, et al. Predicting response to intravesical BCG in high-risk NMIBC using an artificial intelligence-powered pathology assay: development and validation in an international 12-center cohort. J Urol 2025;213:192–204.

[2] He K, Zhang X, Ren S, Sun J. Deep residual learning for image recognition. 2016 IEEE Conference on Computer Vision and Pattern Recognition (CVPR); 2016. p. 770–8.

[3] Loshchilov I, Hutter F. Decoupled weight decay regularization. 2017. p. arXiv:1711.05101.

[4] Nimgaonkar V, Krishna V, Krishna V, et al. Development of an artificial intelligence-derived histologic signature associated with adjuvant gemcitabine treatment outcomes in pancreatic cancer. Cell Rep Med 2023;4:101013.

[5] Hanahan D, Weinberg RA. Hallmarks of cancer: the next generation. Cell 2011;144:646–74.

[6] Hsieh FY, Lavori PW. Sample-size calculations for the Cox proportional hazards regression model with nonbinary covariates. Control Clin Trials 2000;21:552–60.
