# Supplementary material for: Presence of an Artificial Intelligence–powered Predictive Biomarker Is Associated with a Poor Response to Intravesical Bacillus Calmette-Guerin but Not to Intravesical Sequential Gemcitabine/Docetaxel in Patients with High-grade Non–muscle-invasive Bladder Cancer
Source: Eur Urol Oncol. Author manuscript; Available in PMC 2026 Feb 16. (PMC12907750; doi:10.1016/j.euo.2025.04.006)
Supplement: Supplemental Materials [file NIHMS2110211-supplement-Supplemental_Materials.zip › 1-s2.0-S2588931125001002-mmc2.docx]

**Supplementary Table 1 – Patient characteristics by treatment received**

| Characteristic | Gem/Doce (*N* = 94) | BCG (*N* = 159) | *p* value |
| --- | --- | --- | --- |
| CHAI biomarker status, *n* (%) |  |  | 0.3 ^a^ |
| Biomarker absent | 61 (65) | 115 (72) |  |
| Biomarker present | 33 (35) | 44 (28) |  |
| Median age (Q1, Q3) | 73 (66, 80) | 75 (66, 81) | 0.9 ^b^ |
| Sex, *n* (%) |  |  | 0.9 ^a^ |
| Female | 17 (18) | 31 (19) |  |
| Male | 77 (82) | 128 (81) |  |
| Race, *n* (%) |  |  | 0.6 ^a^ |
| Non-white | 4 (4.3) | 11 (6.9) |  |
| White | 90 (96) | 148 (93) |  |
| Site, *n* (%) |  |  | 0.056 ^a^ |
| Iowa | 76 (81) | 111 (70) |  |
| Kentucky | 18 (19) | 48 (30) |  |
| Smoking status, *n* (%) |  |  | 0.9 ^a^ |
| Current | 50 (53) | 88 (55) |  |
| Former | 18 (19) | 28 (18) |  |
| Never | 26 (28) | 43 (27) |  |
| Pretreatment tumor pathology, *n* (%) |  |  | 0.3 ^a^ |
| CIS alone | 7 (7.4) | 12 (7.5) |  |
| T1 | 33 (35) | 70 (44) |  |
| T1 + CIS | 13 (14) | 16 (10) |  |
| Ta | 26 (28) | 47 (30) |  |
| Ta + CIS | 15 (16) | 14 (8.8) |  |
| Pretreatment tumor size (cm), *n* (%) |  |  | 0.072 ^a^ |
| ≤3 | 36 (38) | 51 (32) |  |
| >3 | 37 (39) | 85 (53) |  |
| Unknown | 21 (22) | 23 (14) |  |
| Pretreatment CIS-containing tumor, *n* (%) | 35 (37) | 42 (26) | 0.089 ^a^ |
| Pretreatment multifocal, *n* (%) | 29 (31) | 45 (28) | 0.7 ^a^ |
| Pretreatment variant histology, *n* (%) | 10 (11) | 12 (7.5) | 0.5 ^a^ |
| AUA risk group, *n* (%) |  |  | >0.9 ^a^ |
| High | 88 (94) | 148 (93) |  |
| Intermediate | 6 (6.4) | 11 (6.9) |  |
| EAU risk group, *n* (%) |  |  | 0.5 ^a^ |
| Intermediate | 11 (12) | 27 (17) |  |
| High | 69 (73) | 112 (70) |  |
| Very high | 14 (15) | 20 (13) |  |

AUA = American Urological Association; BCG = bacillus Calmette-Guerin; CHAI = Computational Histologic Artificial Intelligence; CIS = carcinoma in situ; EAU = European Association of Urology; Gem/Doce = gemcitabine and docetaxel.

Tabulated patient characteristics are reported in the BCG- and gemcitabine/docetaxel-treated subgroups. Comparisons between groups were made using Fisher’s exact test for categorical variables and Wilcoxon rank sum test for continuous variables.

^a^ Fisher's exact test.

^b^ Wilcoxon rank sum test.
