# Supplementary figures and images for: Presence of an Artificial Intelligence–powered Predictive Biomarker Is Associated with a Poor Response to Intravesical Bacillus Calmette-Guerin but Not to Intravesical Sequential Gemcitabine/Docetaxel in Patients with High-grade Non–muscle-invasive Bladder Cancer
Source: Eur Urol Oncol. Author manuscript; Available in PMC 2026 Feb 16. (PMC12907750; doi:10.1016/j.euo.2025.04.006)

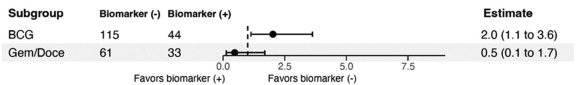

Supplement: S1 MMC3 [file NIHMS2110211-supplement-S1_MMC3.jpg]
